# Microbiome and cancer immunotherapy: a bibliometric analysis

**DOI:** 10.1186/s43046-026-00376-5

**Published:** 2026-06-26

**Authors:** Wenbin Tan

**Affiliations:** Jining Medical University, Jining, China

**Keywords:** Microbiome, Cancer immunotherapy, Bibliometrics, Academic lineage, Knowledge flow, Interdisciplinary research

## Abstract

**Objective:**

To systematically analyze the global research landscape, collaboration patterns, knowledge flow pathways, and frontier trends in the synergistic effects between the microbiome and cancer immunotherapy.

**Methods:**

A systematic bibliometric analysis of publications on the synergistic effects between the microbiome and cancer immunotherapy (2010–2026) was performed using the Web of Science Core Collection. After deduplication, 3,058 publications were analyzed with CiteSpace 6.3.1 (co-citation, keyword burst, timeline), VOSviewer 1.6.19 (co-authorship, co-occurrence networks), and bibliometrix R package 4.4.1 (dual-map overlay, knowledge flow).

**Results:**

Annual publications grew at an average rate of 23.0%, reaching 715 in 2025. China and the United States contributed 60.1% of global output, with MD Anderson Cancer Center, Shanghai Jiao Tong University, and Paris-Saclay University serving as core collaboration hubs. Three major academic lineages (fundamental mechanisms, clinical translation, tumor-specific research) and three knowledge flow pathways (mathematical modeling → molecular genetics; clinical medicine → molecular biology; ecology → molecular biology) were identified, shaping distinct research paradigms. Hotspots evolved from “gut microbiota–ICIs” toward “oral microbiota,” “engineered bacteria,” and “precision prediction.”

**Conclusion:**

The field has transitioned from mechanistic exploration to precision intervention, with multidisciplinary integration and clinical translation as future priorities. The identified knowledge flow pathways and academic lineages provide a framework for understanding the intellectual structure of this rapidly evolving domain.

**Supplementary Information:**

The online version contains supplementary material available at 10.1186/s43046-026-00376-5.

## Introduction

Cancer immunotherapy has revolutionized tumor treatment, with immune checkpoint inhibitors (ICIs) such as programmed cell death protein 1 (PD-1)/programmed death-ligand 1 (PD-L1) and cytotoxic T-lymphocyte-associated protein 4 (CTLA-4) inhibitors becoming standard therapies for multiple malignancies [1–5]. However, only 20–40% of patients achieve long-term benefit from ICIs, showing significant interindividual variability in efficacy [6]. Accumulating evidence confirms that the human microbiome, especially the gut microbiome, modulates host antitumor immune responses to affect ICI efficacy and adverse reactions, emerging as a key breakthrough in overcoming immunotherapy efficacy bottlenecks [7].

In this study, the term “synergistic effects” refers to three interrelated dimensions: (1) the microbiome’s modulation of host antitumor immune responses that enhances ICI therapeutic efficacy; (2) how immunotherapies, in turn, reshape the microbial ecosystem through immune-mediated selection; and (3) the bidirectional metabolic and signaling crosstalk between microbial communities and immune checkpoint pathways that collectively shapes treatment outcomes [8]. This conceptual framework underpins the bibliometric scope of our analysis.

The interplay between the microbiome and cancer immunotherapy has rapidly become an interdisciplinary research frontier, with related literature growing exponentially [9]. However, involving microbiology, immunology, oncology, bioinformatics, and ecology, the research content is fragmented and the knowledge system complex, making it challenging for researchers to quickly grasp the overall landscape and emerging trends.

Several bibliometric studies have examined this field, but they share significant limitations: (a) temporal coverage is mostly limited to pre-2020, missing the rapid expansion during 2021–2025; (b) most rely on a single bibliometric tool (typically VOSviewer alone), lacking the multi-tool triangulation necessary for robust analysis; (c) none have performed academic lineage analysis, which traces intellectual inheritance and the evolution of research traditions; (d) knowledge flow pathways across disciplines remain unidentified, obscuring how different fields contribute to and shape this domain; and (e) frontier detection has been limited to simple keyword frequency analysis, without integrating emergence detection, timeline evolution, and cluster peak landscape analyses [10]. These specific gaps motivate the present study.

Methodologically, bibliometrics has evolved substantially in recent years. Traditional descriptive metrics (publication counts, citation analysis) have been augmented by machine-learning-driven informatics approaches that enable large-scale knowledge mapping [11]. Bibliometric analysis is now applied broadly, from basic molecular biology research to clinical medicine and health policy evaluation [12]. Recent large-scale bibliometric studies have successfully mapped emerging interdisciplinary fields such as large language models in medicine, demonstrating the power of multi-dimensional analytical frameworks [13]. The integration of co-occurrence analysis, clustering algorithms, burst detection, and timeline mapping represents the current state of the art in comprehensive knowledge domain visualization.

Thus, this study selected literature from 2010 to January 2026 and used multiple bibliometric tools (CiteSpace, VOSviewer, and R bibliometrix) to analyze publication trends, collaboration networks, academic lineages, knowledge flow pathways, hotspots, and frontiers. By addressing the aforementioned gaps, this study provides a more comprehensive and temporally extended bibliometric map than existing analyses, offering references for follow-up research, international collaboration, and the standardized development of this field.

## Methods

### Literature search strategy

The Web of Science Core Collection (SCI-E) served as the primary data source, covering January 1, 2010, to January 31, 2026. The search strategy was as follows: TS = (microbiome OR “gut microbiota” OR “intestinal microbiota” OR “oral microbiota” OR “tumor microbiota”) AND TS = (“cancer immunotherapy” OR immunotherapy OR “checkpoint inhibitor” OR PD-1 OR PD-L1 OR CTLA-4).

Screening criteria: (1) Document types: original articles and reviews (excluding conference abstracts, letters, and editorials); (2) Language: English only; (3) Manual exclusion of literature unrelated to synergistic effects; (4) Exclusion of duplicates via Web of Science and manual verification. Ultimately, 3,058 articles were included.

### Data processing and analysis tools

EndNote X9 was used for deduplication, screening, and organization, with basic information extracted and standardized. Multiple tools were applied for comprehensive analysis: (1) CiteSpace 6.3.1 for author collaboration networks, keyword co-occurrence/emergence maps (using Kleinberg’s burst detection algorithm, where burst strength quantifies the intensity of sudden keyword frequency increases relative to baseline), and citation cluster analysis [14]; (2) VOSviewer 1.6.19 for country/institution collaboration network visualization, using the association strength normalization method for keyword clustering, with cluster labels determined automatically by term frequency and relevance scores, then manually reviewed for thematic accuracy [15]; (3) R 4.4.1 (bibliometrix package) for calculating publication metrics and plotting trends; (4) Microsoft Excel 2021 for data organization and table generation.

### Analytical indicators

Core indicators included: (1) Publication trends (annual volume, average annual growth rate); (2) Collaboration networks (country/institution publication volume, collaboration intensity, total link strength, node degree); (3) Core authors (publication volume, collaboration networks, academic lineage); (4) Journal distribution and knowledge flow (publication volume, citations, JCR ranking, dual-map overlays); (5) Research hotspots/frontiers (keyword co-occurrence/clustering/emergence, timeline evolution, cluster peak landscapes); (6) Knowledge base (highly cited papers, cited paper clustering, knowledge module identification).

Ethics statement: This study did not involve human participants or animal experiments; therefore, no ethical approval or informed consent was required.

## Results

### Publication trends

**Fig. 1:**
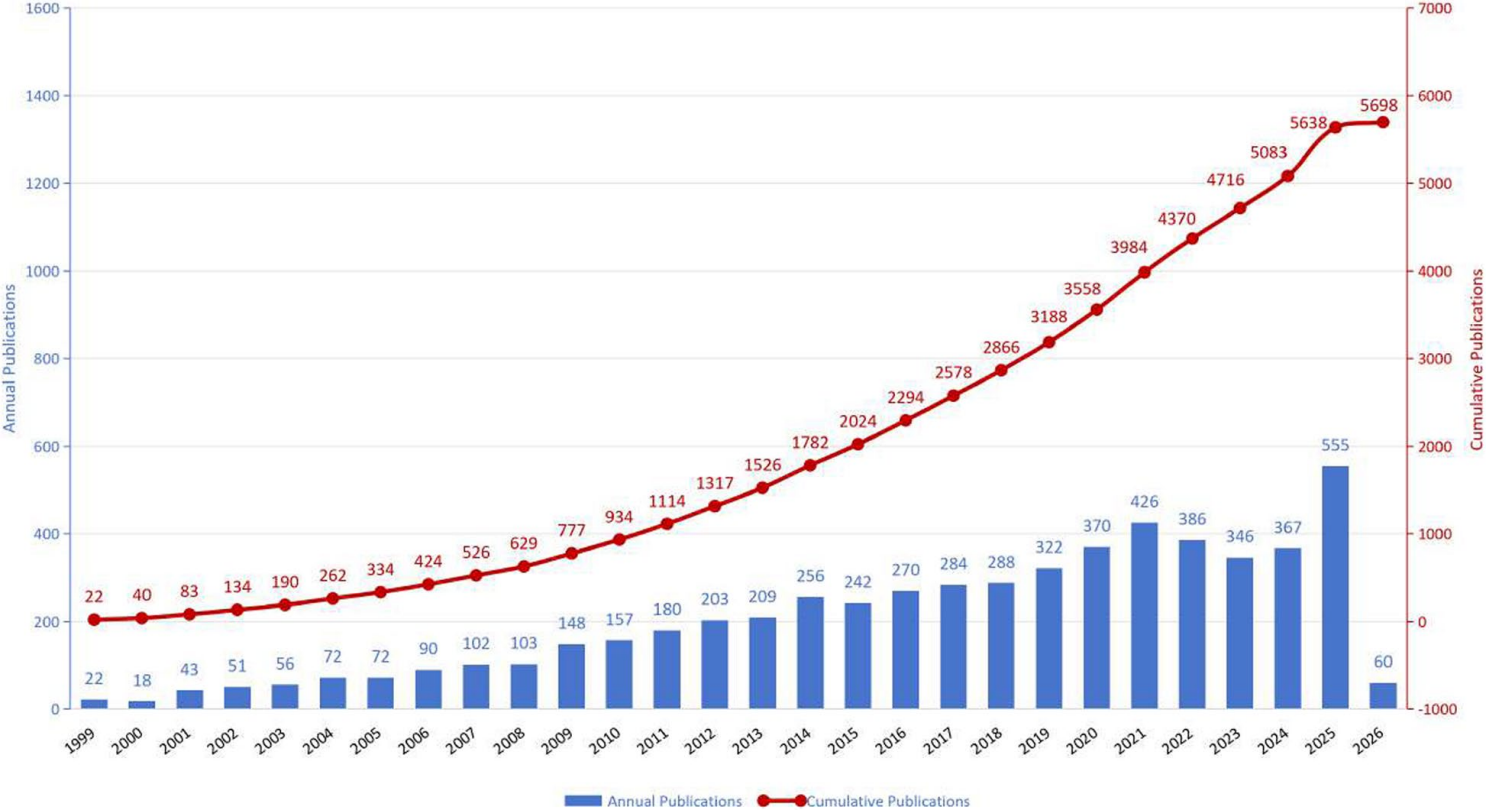
Annual publication volume and cumulative publication volume

Annual publications showed sustained rapid growth from 2010 to 2025, with an exploratory phase followed by a rapid ascent: only one publication in 2010, 255 in 2020, and 715 in 2025. The average annual growth rate was ~ 23.0% from 2020 to 2025, with 2010–2019 as the exploratory phase (38 annual publications on average), 2020–2022 as steady growth (330 annual publications), and 2023–2025 as rapid growth (557 annual publications). The 46.1% surge in 2025 compared to 2024 marks a critical inflection point for accelerated development. The 52 publications in 2026 reflect incomplete data collection (Fig. 1).

This rapid development is closely linked to global cancer immunotherapy breakthroughs; Routy et al.’s 2018 seminal Science study first demonstrated the gut microbiome–PD-1 inhibitor association [16], and advances in multi-omics and bioinformatics technologies have provided technical support for microbiome analysis [17].

### National and institutional collaboration networks

#### Publication distribution by country/region

The 3,058 publications originated from 86 countries and regions. China (1,089, 35.7%) and the United States (749, 24.5%) ranked top two, accounting for ~ 60.1% of global output, as the core research forces. Italy (267, 8.7%), France (141, 4.6%), and Japan (191, 6.3%) followed. Seven of the top ten are developed countries, while China, a developing country, leads in publication volume, reflecting its heavy research investment (Table S1).

#### National/regional collaboration networks

**Fig. 2:**
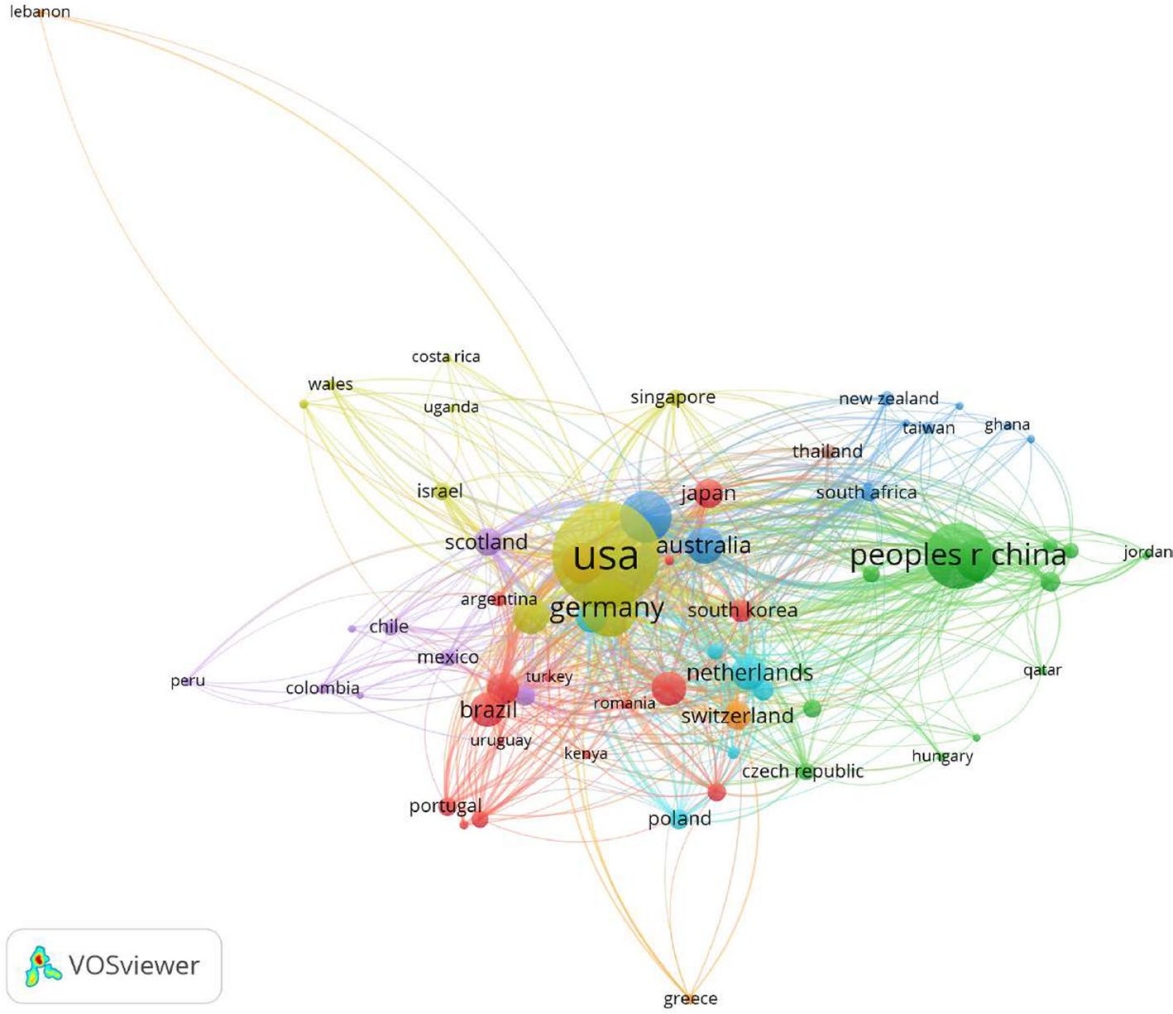
National Collaboration Network. Node size is proportional to publication volume; link thickness represents collaboration intensity (total link strength). Colors indicate collaboration clusters identified by VOSviewer. See Table S1 for quantitative collaboration metrics

The global collaboration network exhibits a “core-periphery” structure, with close ties between China, the United States, and European countries. China and the United States have the strongest collaboration intensity; the US forms a “European–American core circle” with Germany, the UK, and France, while China forms an “East Asian circle” with Japan and South Korea. Australia and Canada maintain ties with core nations, but collaboration among developing countries remains weak, indicating a network imbalance (Fig. 2).

#### Core research institutions

**Fig. 3:**
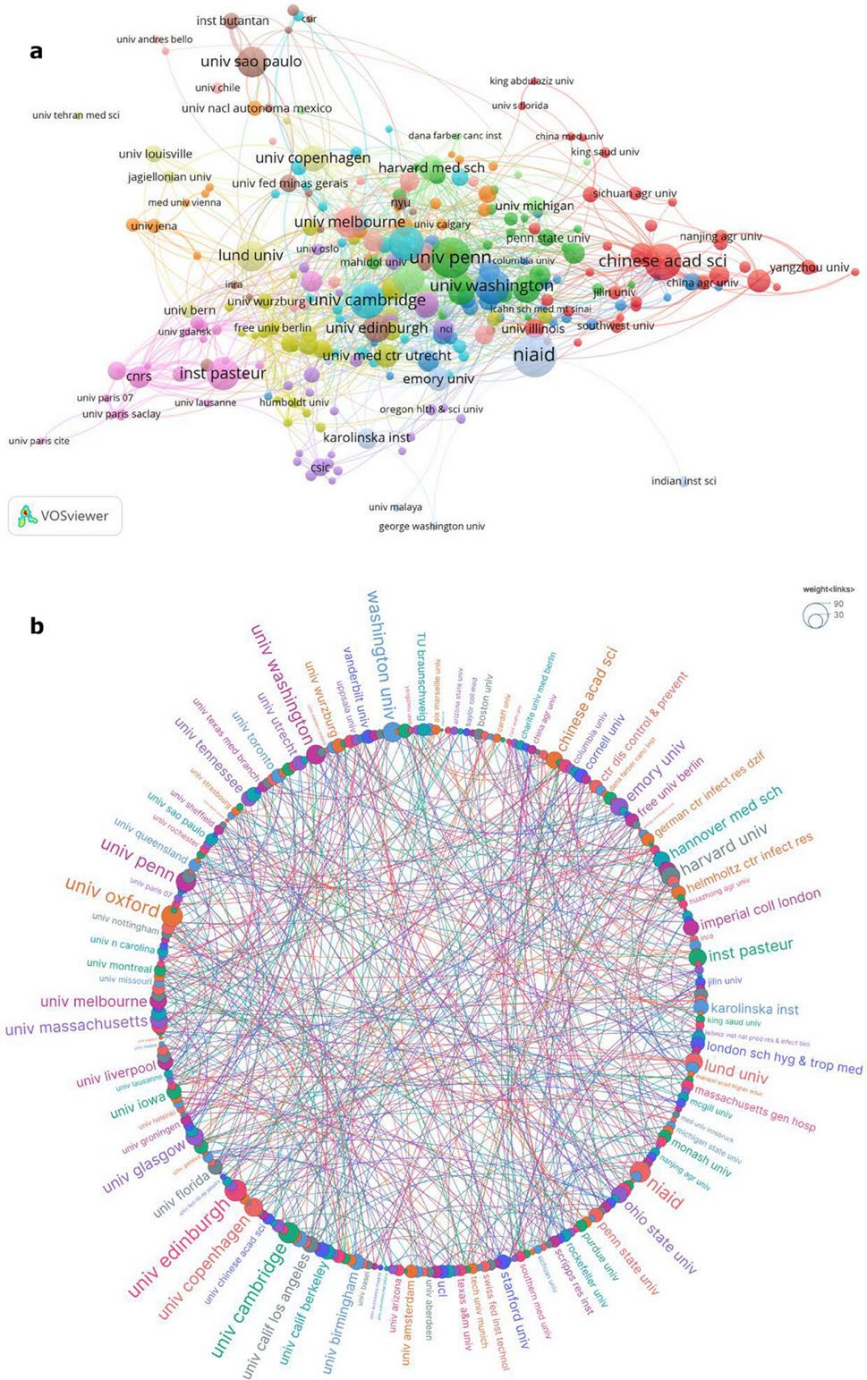
**a** Institutional Collaboration Network – by VosViewer. **b** Institutional Collaboration Network – by Graphica

The top 10 institutions include three US, six Chinese, and one French institution, reflecting the core strength of China and the United States, with China holding a numerical advantage. MD Anderson Cancer Center ranked first (102 papers, 16,695 citations, H-index 62), leading in both output and influence; Shanghai Jiao Tong University was the top Chinese institution (71 papers); and Paris-Saclay University (64 papers, 10,779 citations) demonstrated high research impact. Chinese institutions lag behind top US institutions in citations and H-index, with room for quality improvement. Tight collaboration networks have formed among the top institutions, with MD Anderson Cancer Center, Shanghai Jiao Tong University, and Paris-Saclay University serving as international collaboration hubs (Fig. 3a and b).

### Core author analysis

#### Core author publication distribution

Based on Price’s Law, the core author publication threshold was eight papers, identifying 156 core authors who published 2,456 papers (80.3% of global output), playing a pivotal leadership role. Zitvogel L (France, 44 papers), a founding scholar in gut microbiome-mediated antitumor immunity [18], and Routy B (40 papers), focused on fecal microbiota transplantation (FMT) in immunotherapy [16], ranked top two. Wargo JA (37 papers, 7,166 citations) focused on clinical translation with significant academic influence [19]. Chinese scholar Yu J (19 papers) focused on the gastrointestinal tumor microbiome and immunotherapy, representing China’s academic contribution [20].

#### Core author collaboration networks

**Fig. 4:**
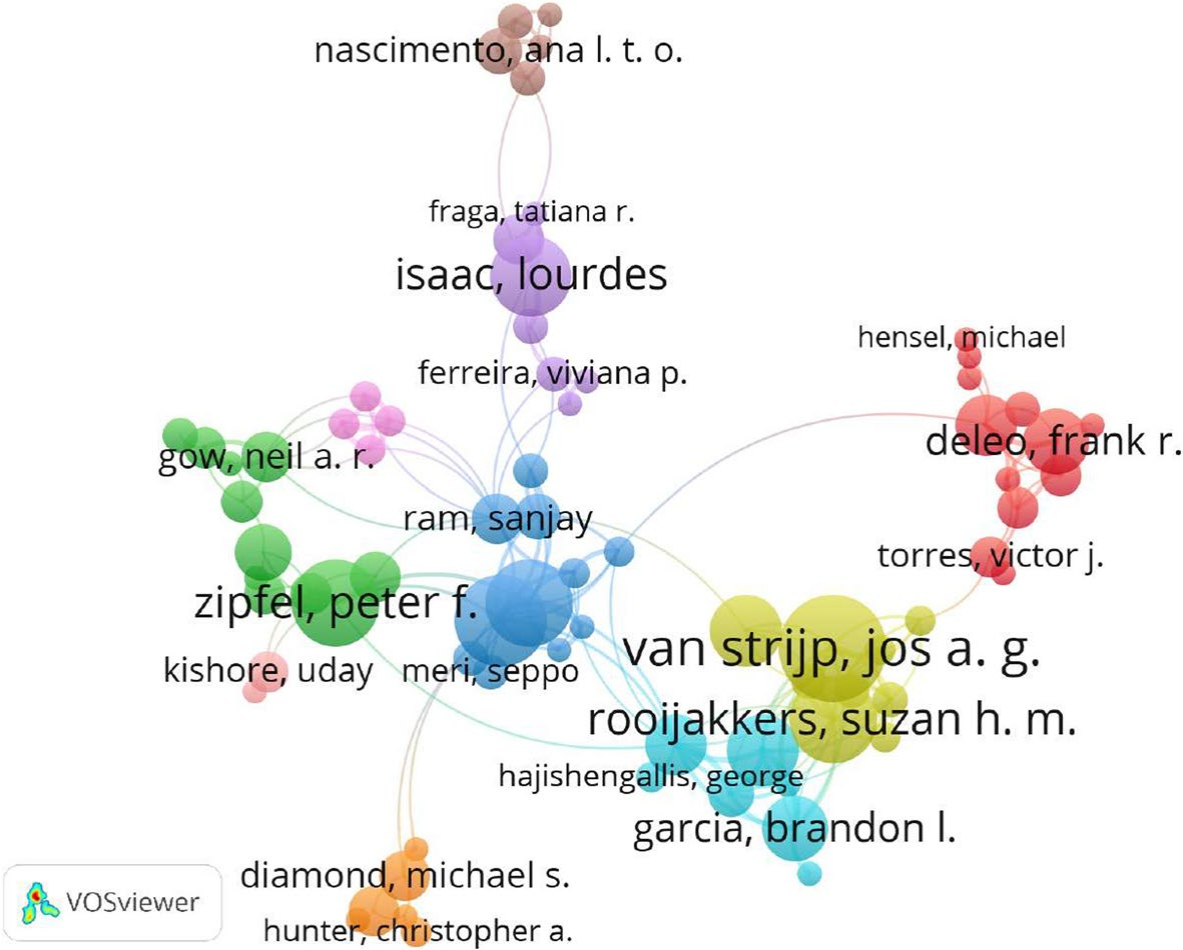
Author collaboration network

Core authors formed multiple tight collaboration clusters with partial overlap: (1) The French cluster (Zitvogel L, Kroemer G) with 31 authors, focusing on gut microbiome–ICI efficacy mechanisms [21]; (2) The US cluster (Wargo JA) with 22 authors, focusing on clinical translational research [22]; (3) The Chinese cluster (Yu J, Wang Ying) with 19 authors, focusing on specific cancer synergistic research and maintaining partial international collaboration [23]. Cross-cluster collaboration is limited, with room for improvement in inter-team cooperation (Fig. 4).

### Academic lineages

**Fig. 5:**
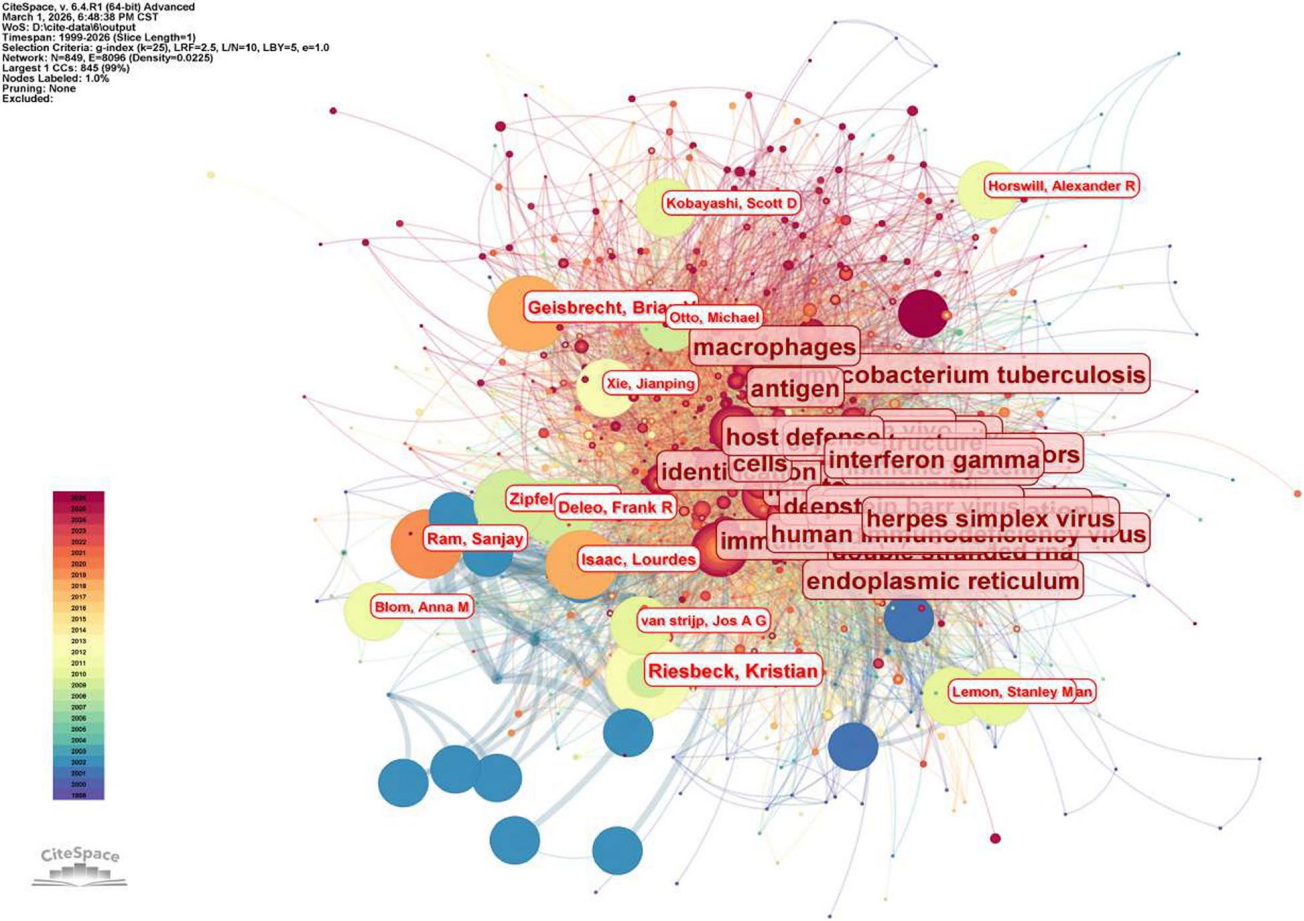
Author-keyword co-occurrence network

Author–keyword co-occurrence analysis (Fig. 5) delineated three major academic lineages: (1) Fundamental Immunological Mechanism Group (Zitvogel L, Kroemer G): Focus on gut microbiome modulation of antitumor immunity and tumor microenvironment reshaping; (2) Clinical Translational Group (Wargo JA, Routy B): Focus on ICI efficacy biomarkers, patient stratification, and FMT clinical intervention [24]; (3) Cancer-Specific Group (Yu J and other Chinese scholars): In-depth exploration of microbiome characteristics of colorectal, liver, lung, and other specific tumors [25].

### Journal distribution and disciplinary knowledge flow

A total of 3,058 papers were published in more than 200 journals. Frontiers in Immunology ranked first (200 articles, Q1), followed by Cancers (142 articles, Q2) and the International Journal of Molecular Sciences (80 articles, Q1/Q2). Eight of the top 10 journals by publication volume are in Q1 (837 articles, 27.4% of the total), indicating the high academic quality of the core literature. Nature Communications had the highest average citations per article (92.1), reflecting strong academic influence.

**Fig. 6:**
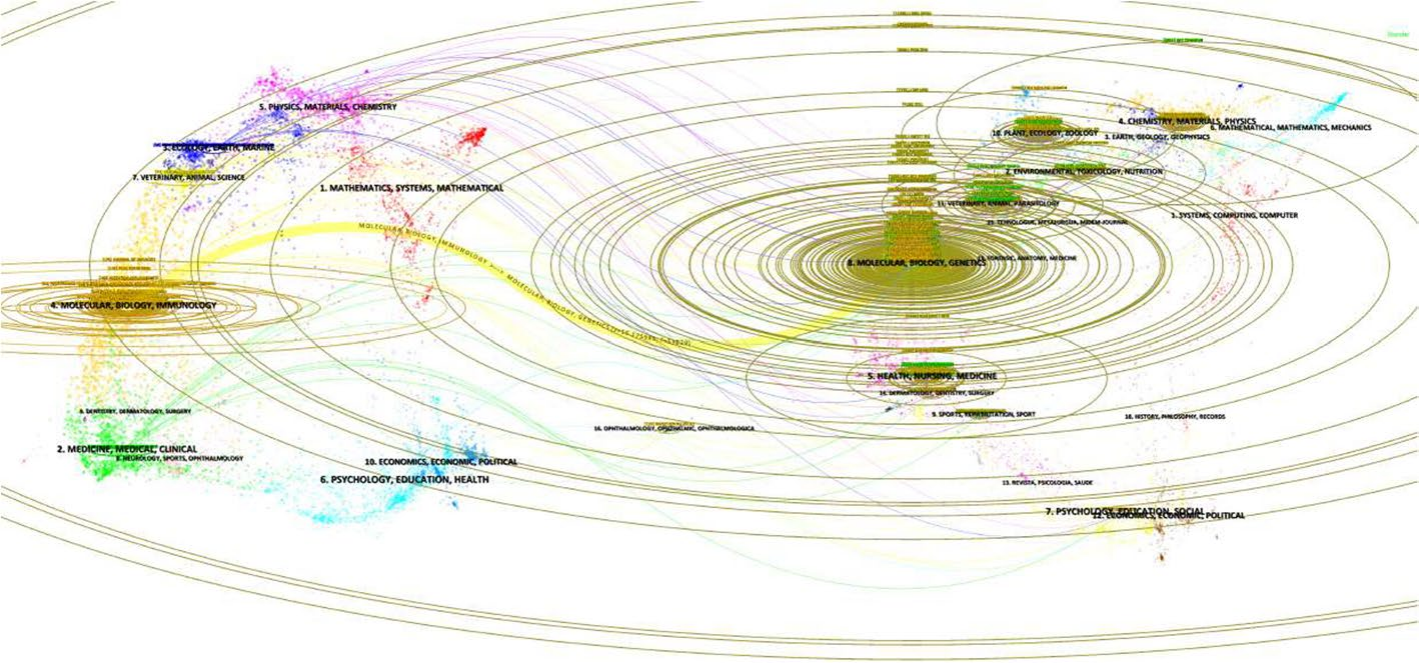
Journal dual-layer overlay map

This field presents an interdisciplinary pattern of “concentrated output and diverse input”: citing journals are mainly in molecular biology and immunology, while cited journals span fundamental biology, clinical medicine, ecology, and bioinformatics. Three core knowledge flow pathways were identified: mathematical modeling → molecular genetics, clinical medicine → molecular biology, and ecology → molecular biology, forming an integrated system with multi-omics as tools, ecological theory as a framework, and clinical translation as the goal (Fig. 6).

#### From knowledge flows to research paradigms

The three knowledge flow pathways identified above have each shaped distinct research paradigms that define this field:

1. Systems Immunology Paradigm. The mathematical modeling → molecular genetics pathway reflects the emergence of a systems-level approach to understanding how the microbiome modulates antitumor immunity. This paradigm treats the host–microbiome–immune axis as an integrated system in which computational and mathematical models generate testable molecular hypotheses, consistent with Kuhn’s concept of paradigm-driven normal science [26].
2. Clinical-to-Basic Translational Paradigm. The clinical medicine → molecular biology pathway represents a bidirectional translational paradigm wherein clinical observations of differential immunotherapy responses across patients with distinct microbiome profiles drive mechanistic molecular investigations, which in turn inform patient stratification and microbiota-targeted intervention strategies.
3. Microbial Ecology Paradigm. The ecology → molecular biology pathway reflects the adoption of ecological frameworks—including community assembly, niche theory, and keystone species concepts—to interpret tumor and gut microbial communities, as articulated in the “microbially conscious” framework of cancer biology [27].

These three paradigms collectively form the intellectual infrastructure of microbiome–cancer immunotherapy research, and their identification provides a novel framework for understanding the field’s evolution.

### Research hotspots and frontier evolution

#### Keyword co-occurrence and clustering

**Fig. 7:**
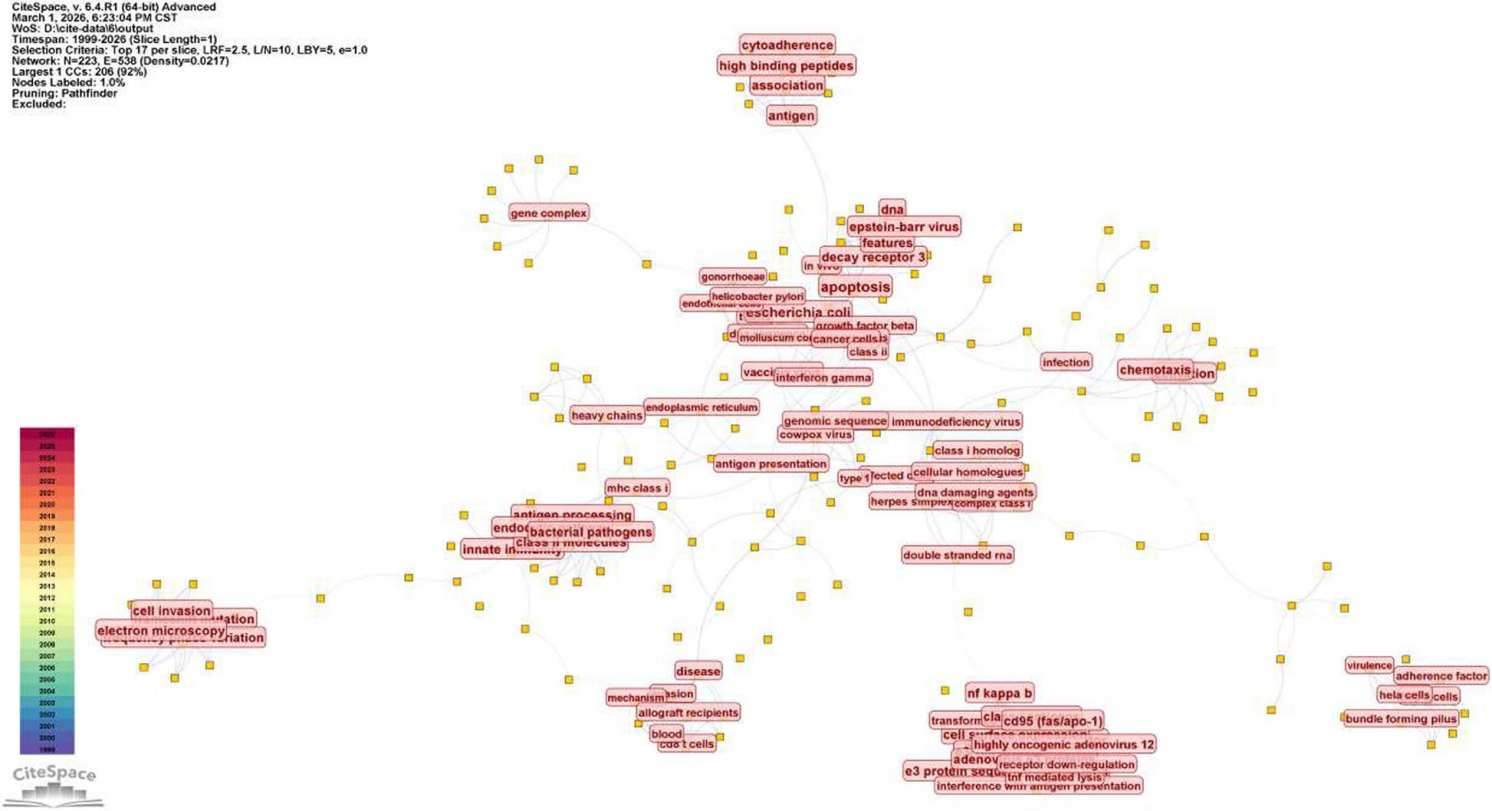
Keyword Co-occurrence Map. Nodes represent keywords (size proportional to frequency); links represent co-occurrence relationships; colors indicate clusters. See Table S2 for quantitative keyword metrics

**Fig. 8:**
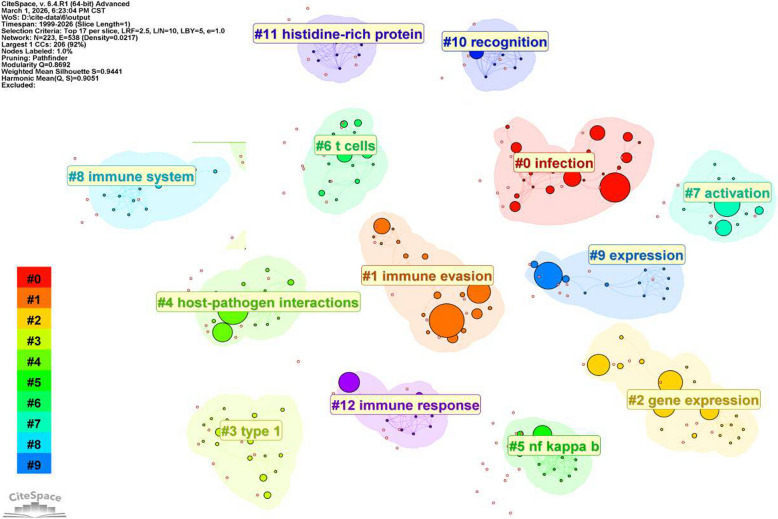
Keyword clustering map

Keyword co-occurrence analysis identified 156 core keywords (≥ 20 occurrences) that formed 14 major clusters (Figs. 7 and 8). The top research hotspots included tumor microenvironment and immune regulation (28.5% of keyword frequency), aryl hydrocarbon receptors and microbial metabolism, colorectal cancer and microbiome intervention, and ICI efficacy mechanisms. Among these, “gut microbiota” showed the strongest co-occurrence with “immunotherapy” and “immune checkpoint inhibitors,” serving as the central research focus.

Specific high-frequency keywords and their network metrics (betweenness centrality, calculated by CiteSpace) included:

- PD-1 (frequency: 389, centrality: 0.19, Cluster 2: Immunotherapy),
- PD-L1 (298, 0.16, Cluster 2),
- CTLA-4 (267, 0.14, Cluster 2),
- short-chain fatty acids (205, 0.13, Cluster 11: Metabolites),
- immune-related adverse events (54, 0.04, Cluster 16: irAEs),
- tumor-infiltrating lymphocytes (28, 0.05, Cluster 3: TME regulation).

These keywords reflect the field’s focus on key immunotherapeutic targets, microbial metabolites, treatment-related adverse events, and tumor microenvironment regulation. Cluster names were determined by CiteSpace’s log-likelihood ratio (LLR) algorithm, which extracts representative noun phrases from citing papers, and were manually verified for semantic coherence (see Fig. 8 for cluster visualization).

#### Keyword emergence

**Fig. 9:**
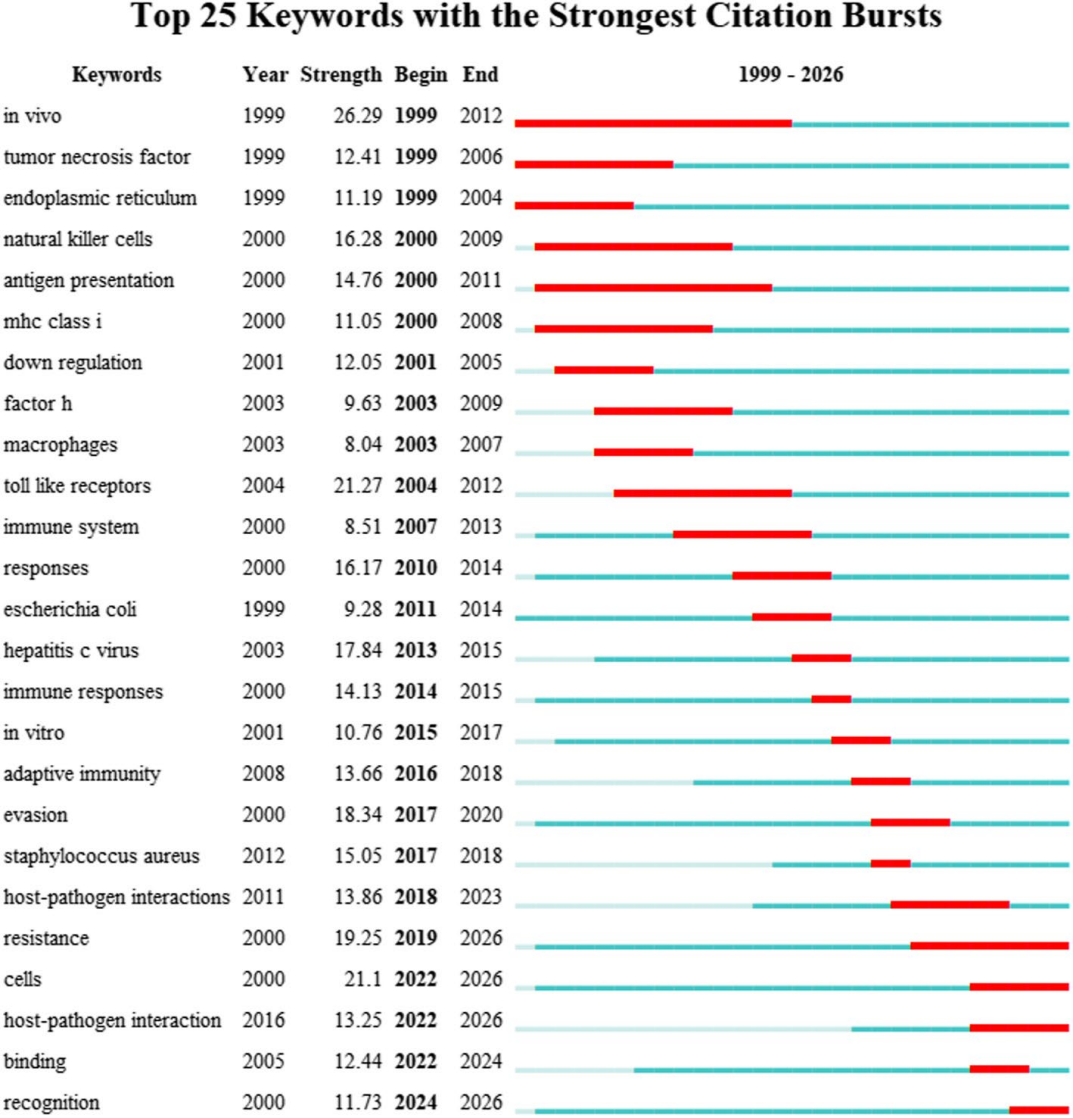
Keyword emergence list

Twenty-five emerging keywords were identified using CiteSpace’s burst detection algorithm (Kleinberg’s algorithm), where burst strength quantifies the intensity of sudden keyword frequency increases relative to baseline occurrence. Based on the temporal clustering of burst start years, research frontiers were divided into three distinct phases (Fig. 9):

Phase I (2013–2018): Fundamental Mechanisms — Early bursts centered on immunological fundamentals, including “dendritic cells,” “CTLA-4,” and “PD-1 blockade,” reflecting initial exploration of the immunological basis of microbiome–immunotherapy interactions [28, 29].

Phase II (2017–2021): Clinical Translation — Burst keywords shifted toward clinical applications, including “tumor-infiltrating lymphocytes” and “nivolumab,” marking the transition from laboratory findings to therapeutic contexts [30, 31].

Phase III (2019–2025): Precision Applications — Recent bursts reflect movement toward targeted, personalized strategies: “oral microbiota,” “engineered bacteria,” “tyrosine kinase inhibitors,” and “precision efficacy prediction” [32, 33]. The emergence of Salmonella Typhimurium as a keyword reflects the growing interest in bacteriotherapy as a cancer treatment modality.

Phase boundaries were determined by the median burst start year of keywords within each thematic cluster, with overlapping years reflecting the gradual, non-abrupt nature of research priority transitions.

#### Timeline and timezone evolution

**Fig. 10:**
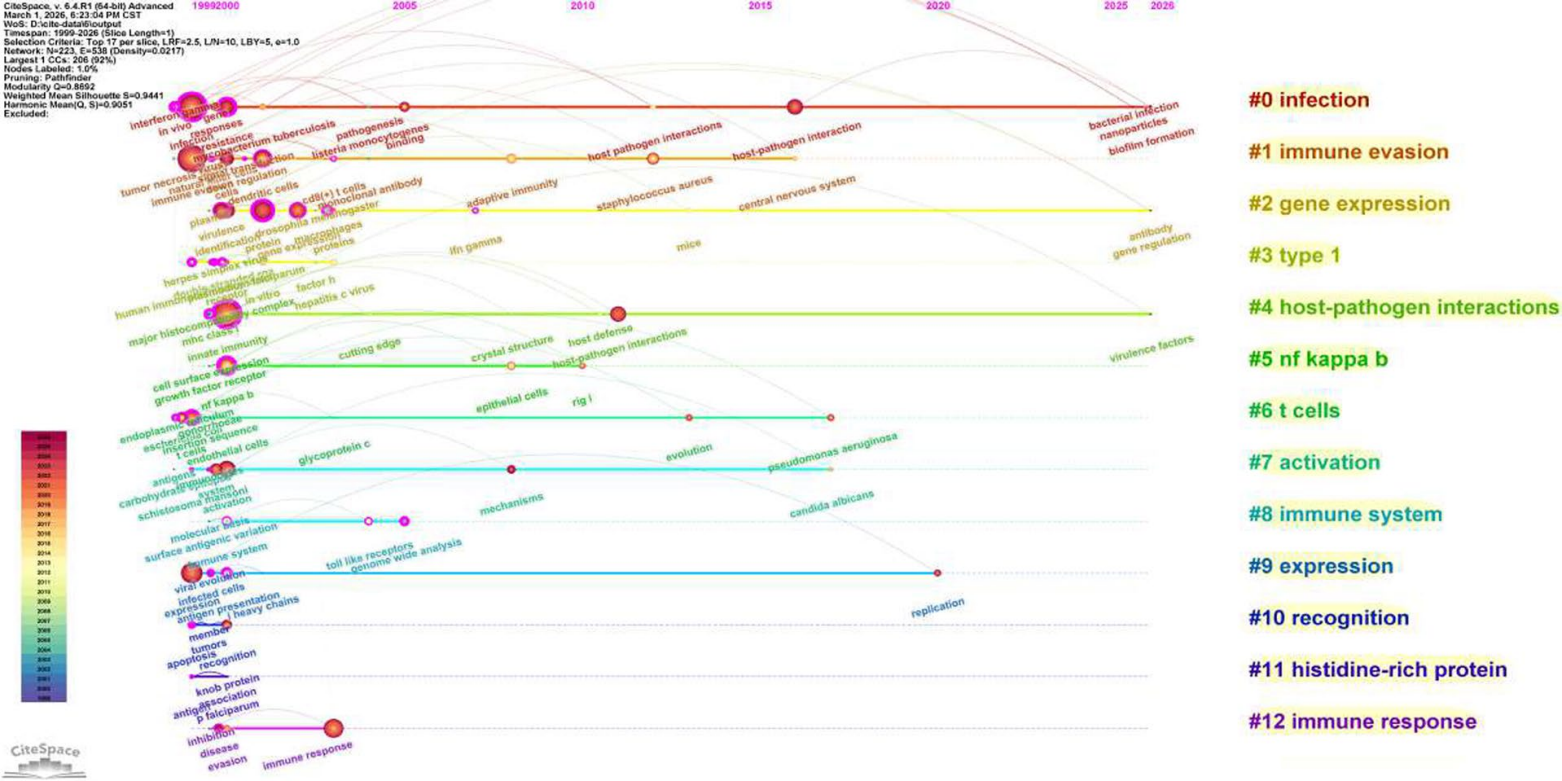
Keyword Timeline Map. Horizontal axis: publication years. Each horizontal line: a keyword cluster (labeled on the right). Nodes: individual keywords (size proportional to frequency). Node position on timeline: median year of keyword occurrence. Lines extending further right indicate clusters remaining active. See Table S3 for cluster-level metrics (silhouette score, size, mean year, top keywords)

Figure 10 (Keyword Timeline Map): This visualization depicts the temporal distribution of keywords within each cluster. The horizontal axis represents publication years; each horizontal line represents a keyword cluster (labeled on the right); nodes on each line represent individual keywords, with node size proportional to occurrence frequency; the node’s position on the timeline indicates the median year of keyword appearance; lines extending further right indicate clusters remaining active in recent years.

**Fig. 11:**
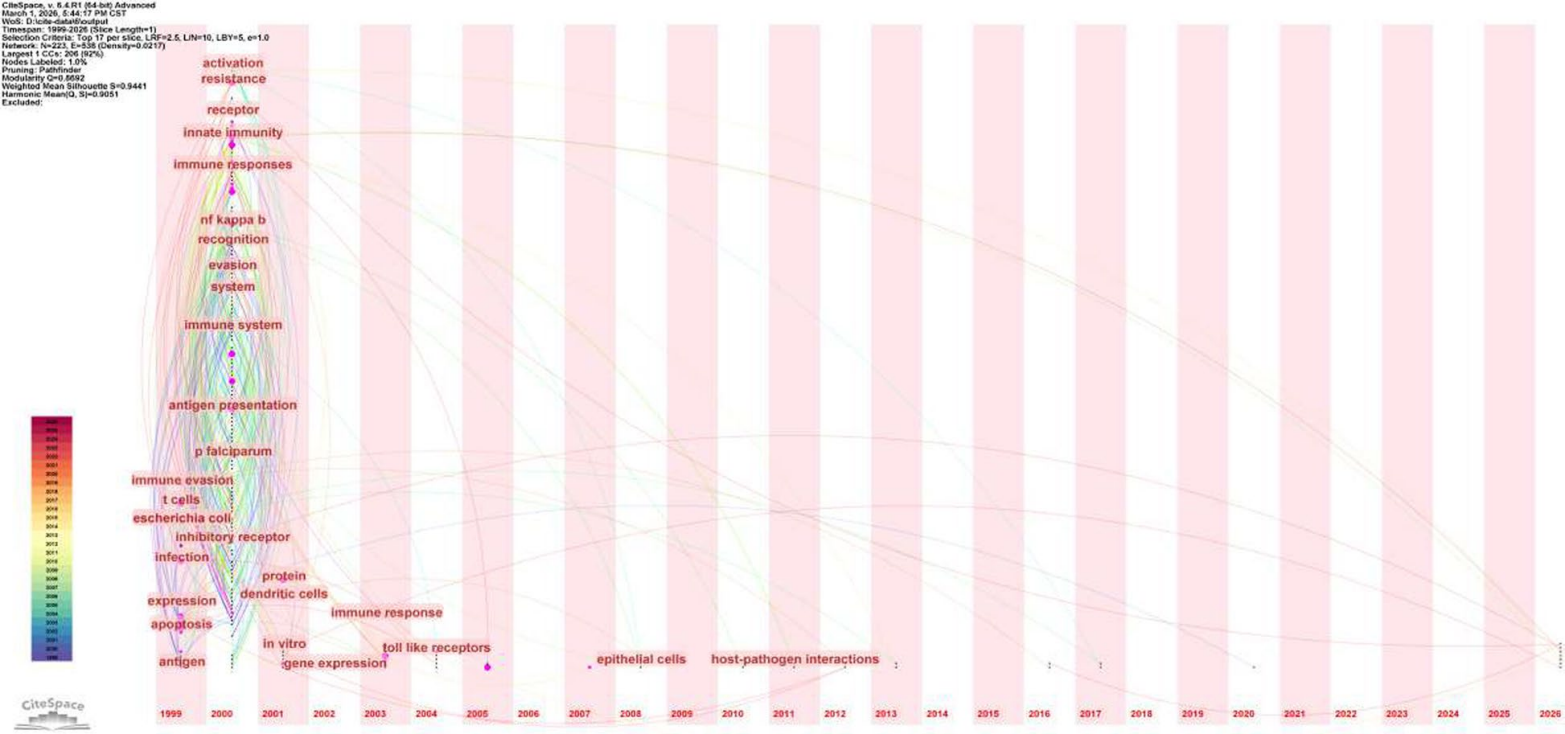
Keyword Time Zone Evolution Map. Each column: one year. Nodes: keywords placed in the year of first significant occurrence. Connecting lines: co-occurrence relationships. Node size: cumulative frequency. Red rings: burst detection status. See Table S4 for yearly keyword statistics

Figure 11 (Keyword Time Zone Evolution Map): Each column represents one year; nodes are keywords placed in the year of first significant occurrence; connecting lines between nodes indicate co-occurrence relationships; node size reflects cumulative frequency; red rings indicate burst detection status.

**Fig. 12:**
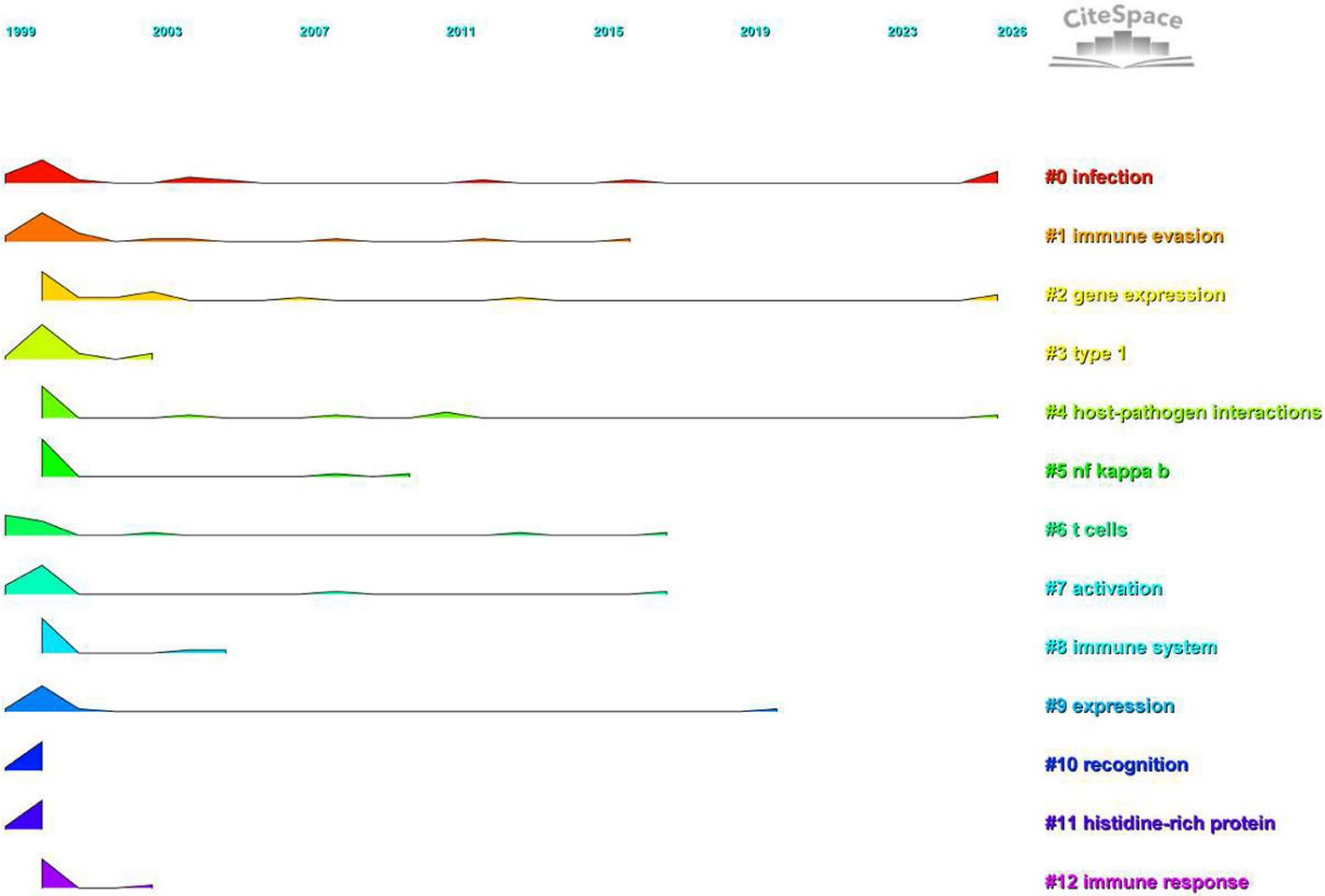
Keyword Cluster Peak Landscape Map. Mountain-like visualization of cluster temporal prominence. Peak height: cluster activity intensity (citation frequency within cluster). Peak width: active duration in years. Colors: cluster differentiation. See Table S5 for peak year, peak citation count, and active period for each cluster

Figure 12 (Keyword Cluster Peak Landscape Map): The mountain-like landscape visualizes the temporal prominence of each keyword cluster. Peak height represents cluster activity intensity (measured by citation frequency within the cluster); peak width represents the cluster’s active duration in years; colors differentiate clusters.

### Highly cited literature and knowledge base

Papers with ≥ 200 citations were defined as highly cited, with 156 identified (5.1% of total), mainly published in Science, Nature, and Cell. Routy et al.’s 2018 Science study (4,218 citations) [16], Gopalakrishnan et al.’s 2018 Science study (3,592 citations) [19], and Vétizou et al.’s 2015 Science study (2,721 citations) [18] form the core knowledge foundation, demonstrating the microbiome–ICI association and CTLA-4 inhibitor modulation by gut microbiota.

The cited references were categorized into six knowledge modules through a two-step process: (1) Citation clustering: Using CiteSpace’s cited-reference clustering function with the log-likelihood ratio (LLR) algorithm, highly cited papers were grouped based on shared citation patterns. (2) Module definition and naming: Each cluster was independently inspected by two researchers who reviewed the titles and abstracts of the top 10 most-cited papers within each cluster. Cluster labels were assigned by consensus using both LLR-generated keywords and the researchers’ content analysis; a third researcher mediated any discrepancies.

The resulting six knowledge modules are: (1) gut microbiome–PD-1/PD-L1 association; (2) microbial intervention strategies (FMT, probiotics, antibiotics); (3) tumor microenvironment immunomodulation; (4) cancer-type-specific microbiome research; (5) multi-omics and bioinformatics applications; and (6) immune-related adverse event regulation. These modules establish a knowledge system centered on the “gut microbiome–immune checkpoint inhibitors–antitumor immunity” axis, encompassing basic mechanisms, clinical applications, and technical methods.

## Discussion

This study provides the most comprehensive bibliometric analysis to date of the microbiome–cancer immunotherapy field, encompassing 3,058 publications over a 16-year period (2010–2026) with multi-tool analytical integration. Beyond mapping publication trends and collaboration networks, we identified three academic lineages, three knowledge flow pathways, and their corresponding research paradigms—dimensions absent from prior bibliometric studies [10].

The dominance of China and the United States (60.1% of global output) reflects not only the magnitude of research investment but also structural factors in the global research ecosystem. China’s publication volume leadership can be attributed to large-scale government funding initiatives (e.g., National Natural Science Foundation microbiome priority areas) and institutional publication incentives that reward output volume. However, the citation gap—the US H-index of 92 versus China’s 75—warrants scrutiny. This disparity may stem from several factors: the US groups’ first-mover advantage in producing foundational discoveries (e.g., the 2015–2018 Science papers from the Zitvogel, Wargo, and Gopalakrishnan groups); lower participation of Chinese researchers in high-impact international multicenter collaborations; and a higher proportion of descriptive or correlational studies relative to mechanistic investigations in the Chinese literature. Addressing this quality gap requires not merely increased funding but structural reforms in research evaluation that prioritize originality over publication counts [34].

The concentration of core literature in Q1 journals is a double-edged signal. While it indicates high academic standards, it also raises the question of publication bias: negative-result or null-finding microbiome–immunotherapy studies may face disproportionate barriers to publication in high-impact venues, potentially skewing the evidence base toward positive associations. The dominance of oncology and immunology journals further suggests that microbiological and ecological perspectives remain underrepresented in the core literature, despite the importance of the knowledge flow pathways we identified from ecology to molecular biology.

Several notable research gaps emerge from our analysis. First, the gut bacteriome overwhelmingly dominates the literature, with fungal (mycobiome) and viral (virome) components receiving disproportionately little attention despite their known immunomodulatory roles. Second, geographic representation is highly skewed toward North America, Europe, and East Asia, with Africa, South America, and South Asia contributing fewer than 3% of publications combined—a critical deficiency given that microbiome composition varies substantially across populations. Third, pediatric immunotherapy contexts remain almost entirely unexplored in microbiome research, despite the distinct immune maturation profiles and microbiota composition in children and geographies. Fourth, the majority of studies remain correlational; randomized intervention trials (e.g., FMT, defined probiotic consortia) are scarce, limiting the translational impact of mechanistic discoveries. Finally, the lack of standardized microbiome analysis pipelines (from sample collection to bioinformatic processing) hinders cross-study comparability and meta-analytic synthesis [25].

The three knowledge flow pathways we identified are not merely descriptive patterns; they represent the intellectual infrastructure that has shaped the field’s evolution. The mathematical modeling → molecular genetics pathway gave rise to predictive, systems-level approaches to microbiome–ICI interactions, enabling the recent surge in machine learning-based biomarker discovery. The clinical medicine → molecular biology pathway established the translational feedback loop that distinguishes this field from purely basic microbiome research: clinical observations drive mechanistic inquiry, which in turn informs trial design. The ecology → molecular biology pathway imported conceptual frameworks—keystone species, community resilience, niche construction—that reframe therapeutic intervention as ecosystem engineering rather than single-pathway targeting. These three paradigms are now converging: emerging studies combine ecological modeling with clinical data and molecular validation, exemplified by recent work integrating microbial community network analysis with ICI response prediction [35].

The three academic lineages we delineated—fundamental mechanisms, clinical translation, and tumor-specific research—exhibit complementary strengths but limited cross-lineage collaboration. This siloing may slow translational progress: mechanistic discoveries from the Zitvogel/Kroemer group require clinical validation, while clinical observations from the Wargo group need mechanistic elucidation. Strengthening cross-lineage collaboration, particularly through shared data platforms and standardized protocols, would accelerate the bench-to-bedside pipeline.

The emergence of oral microbiota, engineered bacteria, and precision prediction as Phase III frontiers (2019–2025) signals a paradigm shift from broad microbiome characterization to targeted intervention. Engineered bacteria—such as attenuated Salmonella Typhimurium strains designed to deliver tumor antigens or immunomodulatory molecules—represent a convergence of synthetic biology and immunotherapy. Oral microbiota research expands the anatomical scope of microbiome–cancer investigation beyond the gut, with implications for head and neck cancers and systemic immune modulation. Precision prediction efforts, increasingly leveraging machine learning on multi-omics datasets, aim to identify which patients will benefit from specific microbiome-modulating interventions, moving the field toward personalized medicine.

### Limitations

This study has several limitations: (1) Only the Web of Science Core Collection was used; although WoS is the most widely used database for bibliometric analysis, excluding PubMed, Scopus, and Embase may introduce database coverage bias, particularly for clinical studies indexed preferentially in PubMed. (2) Restriction to English-language literature insufficiently captures contributions from non-English-speaking countries, where significant microbiome research is published in regional languages (e.g., Chinese, Japanese, French). (3) The analysis focused on quantitative bibliometric indicators and did not perform in-depth content mining (e.g., full-text analysis of mechanistic claims), which limits the granularity of our thematic classification. (4) Synonym merging for keywords was performed manually and may be incomplete, slightly affecting clustering precision. (5) Bibliometric indicators (citations, H-index) are subject to temporal bias, favoring older publications; the rapid growth of this field means that recent high-quality studies may be underrepresented in citation-based metrics. (6) Data collection for 2026 is incomplete (cut-off: January 31, 2026), and the apparent decline in publications is an artifact of partial-year coverage.

### Future directions

Based on the trends and gaps identified, future research should prioritize: (1) investigating microbiota regulatory mechanisms with a focus on precisely identifying core functional strains (e.g., Akkermansia muciniphila phylogroups) and clarifying their molecular targets to enable the development of standardized therapeutic formulations; (2) conducting personalized FMT and probiotic clinical trials stratified by patient microbiome profiles, tumor type, and ICI regimen, with rigorous randomized controlled designs; (3) deepening international collaboration with an emphasis on original, mechanistic breakthroughs rather than incremental descriptive studies, and expanding research participation from underrepresented regions; (4) integrating multi-omics, single-cell sequencing, spatial transcriptomics, and artificial intelligence to decipher the complex host–microbiome–immunity interactome and discover novel therapeutic targets; and (5) systematically investigating the role of the microbiome in modulating immune-related adverse events (e.g., colitis, pneumonitis), with the goal of developing probiotic-based, dietary, or antibiotic interventions and establishing predictive systems for toxicity risk stratification.

## Conclusion

This bibliometric analysis reveals that the microbiome–cancer immunotherapy field has entered a phase of accelerated development and deep interdisciplinary integration. The sustained 23.0% annual growth rate and the 2025 inflection point suggest that research activity will continue to intensify. The identification of three academic lineages and three knowledge flow pathways (mathematical modeling → molecular genetics, clinical medicine → molecular biology, ecology → molecular biology) provides a novel framework for understanding the intellectual structure of this domain—one that goes beyond simple publication metrics to reveal how different disciplines have converged to shape current research paradigms.

The field’s evolution from fundamental immunological mechanisms toward precision microbial interventions reflects broader trends in biomedicine: the shift from one-size-fits-all approaches to personalized, multi-modal therapeutic strategies. However, realizing the clinical promise of microbiome modulation in cancer immunotherapy requires addressing the identified gaps—particularly the paucity of randomized intervention trials, the geographic imbalance in research participation, and the need for standardized analytical frameworks.

With continued multidisciplinary collaboration, technological advances in multi-omics and artificial intelligence, and a concerted effort to bridge basic discovery with clinical application, microbiome-informed strategies have the potential to become integral components of cancer immunotherapy, ultimately improving treatment outcomes and patient survival.

## Supplementary Information

Supplementary Material 1: Table S1. Key national collaboration network metrics for the top 15 countries. Table S2. Top 30 core keywords with frequency, betweenness centrality, and cluster assignment. Table S3. Keyword timeline cluster metrics (Fig. 10): silhouette score, cluster size, mean publication year, and top 5 representative keywords for each cluster. Table S4. Keyword time zone evolution statistics (Fig. 11): yearly keyword counts and top 5 newly appearing keywords per year. Table S5. Keyword cluster peak landscape metrics (Fig. 12): peak year, peak citation count, and active period for each cluster.

## Data Availability

No datasets were generated or analysed during the current study.

## Abbreviations

CTLA-4: Cytotoxic T-lymphocyte-associated protein 4
FMT: Fecal microbiota transplantation
ICI: Immune checkpoint inhibitor
JCR: Journal Citation Reports
LLR: Log-likelihood ratio
PD-1: Programmed cell death protein 1
PD-L1: Programmed death-ligand 1
TLS: Total link strength
