# Supplementary material for: Microbiome and cancer immunotherapy: a bibliometric analysis
Source: J Egypt Natl Canc Inst. 2026 Jun 26;38:38. doi: 10.1186/s43046-026-00376-5 (PMC13309577; doi:10.1186/s43046-026-00376-5)
Supplement: Supplementary file 1 — Supplementary Material 1: Table S1. Key national collaboration network metrics for the top 15 countries. Table S2. Top 30 core keywords with frequency, betweenness centrality, and cluster assignment. Table S3. Keyword timeline cluster metrics (Fig. 10): silhouette score, cluster size, mean publication year, and top 5 representative keywords for each cluster. Table S4. Keyword time zone evolution statistics (Fig. 11): yearly keyword counts and top 5 newly appearing keywords per year. Table S5. Keyword cluster peak landscape metrics (Fig. 12): peak year, peak citation count, and active period for each cluster. [file 43046_2026_376_MOESM1_ESM.docx]

**Table S1. Key National Collaboration Network Metrics for the Top 15 Countries**

| **Rank** | **Country** | **Publications** | **Share (%)** | **Links** | **Total Link Strength** | **Citations** | **Avg Citations** |
| --- | --- | --- | --- | --- | --- | --- | --- |
| 1 | China | 1,089 | 35.7 | 44 | 290 | 36,678 | 33.7 |
| 2 | USA | 749 | 24.5 | 65 | 621 | 72,214 | 96.4 |
| 3 | Italy | 267 | 8.7 | 46 | 248 | 15,071 | 56.4 |
| 4 | Japan | 191 | 6.3 | 35 | 132 | 10,774 | 56.4 |
| 5 | Germany | 162 | 5.3 | 44 | 254 | 9,817 | 60.6 |
| 6 | France | 141 | 4.6 | 36 | 185 | 7,619 | 54.0 |
| 7 | UK | 131 | 4.3 | 49 | 266 | 8,415 | 64.2 |
| 8 | South Korea | 103 | 3.4 | 24 | 72 | 4,514 | 43.8 |
| 9 | Canada | 95 | 3.1 | 35 | 176 | 8,006 | 84.3 |
| 10 | Australia | 83 | 2.7 | 28 | 120 | 5,709 | 68.8 |
| 11 | Netherlands | 79 | 2.6 | 30 | 153 | 4,289 | 54.3 |
| 12 | Spain | 73 | 2.4 | 28 | 119 | 3,412 | 46.7 |
| 13 | India | 65 | 2.1 | 20 | 67 | 2,546 | 39.2 |
| 14 | Sweden | 52 | 1.7 | 26 | 93 | 3,019 | 58.1 |
| 15 | Brazil | 48 | 1.6 | 19 | 54 | 1,876 | 39.1 |

**Table S2. Top 30 Core Keywords with Frequency, Betweenness Centrality, and Cluster Assignment**

| **Rank** | **Keyword** | **Frequency** | **Centrality** | **Cluster** |
| --- | --- | --- | --- | --- |
| 1 | gut microbiota | 1,247 | 0.32 | Cluster 1: Gut-ICI axis |
| 2 | immunotherapy | 892 | 0.28 | Cluster 2: Immunotherapy |
| 3 | tumor microenvironment | 621 | 0.24 | Cluster 2: Immunotherapy |
| 4 | fecal microbiota transplantation | 456 | 0.21 | Cluster 1: Gut-ICI axis |
| 5 | short-chain fatty acids | 398 | 0.19 | Cluster 1: Gut-ICI axis |
| 6 | probiotics | 367 | 0.17 | Cluster 1: Gut-ICI axis |
| 7 | PD-L1 | 354 | 0.15 | Cluster 2: Immunotherapy |
| 8 | colorectal cancer | 312 | 0.14 | Cluster 3: CRC microbiome |
| 9 | biomarkers | 298 | 0.13 | Cluster 4: Prediction |
| 10 | dysbiosis | 285 | 0.12 | Cluster 1: Gut-ICI axis |
| 11 | T cells | 274 | 0.11 | Cluster 2: Immunotherapy |
| 12 | immune checkpoint inhibitors | 268 | 0.10 | Cluster 2: Immunotherapy |
| 13 | antibiotics | 254 | 0.09 | Cluster 1: Gut-ICI axis |
| 14 | mouse model | 241 | 0.09 | Cluster 0: Models |
| 15 | diet | 235 | 0.08 | Cluster 1: Gut-ICI axis |
| 16 | inflammation | 228 | 0.08 | Cluster 4: Prediction |
| 17 | melanoma | 219 | 0.07 | Cluster 2: Immunotherapy |
| 18 | diversity | 211 | 0.07 | Cluster 1: Gut-ICI axis |
| 19 | metabolism | 203 | 0.07 | Cluster 4: Prediction |
| 20 | Bifidobacterium | 196 | 0.06 | Cluster 1: Gut-ICI axis |
| 21 | metabolites | 189 | 0.06 | Cluster 4: Prediction |
| 22 | survival | 181 | 0.06 | Cluster 4: Prediction |
| 23 | lung cancer | 174 | 0.05 | Cluster 3: CRC microbiome |
| 24 | butyrate | 168 | 0.05 | Cluster 1: Gut-ICI axis |
| 25 | tumor mutational burden | 162 | 0.05 | Cluster 2: Immunotherapy |
| 26 | combination therapy | 156 | 0.05 | Cluster 2: Immunotherapy |
| 27 | CTLA-4 | 149 | 0.04 | Cluster 2: Immunotherapy |
| 28 | Akkermansia muciniphila | 143 | 0.04 | Cluster 1: Gut-ICI axis |
| 29 | immune cells | 138 | 0.04 | Cluster 2: Immunotherapy |
| 30 | tumor-associated macrophages | 132 | 0.04 | Cluster 2: Immunotherapy |

**Table S3. Keyword Timeline Cluster Metrics (Supporting Figure 10)**

*Modularity Q = 0.7946; weighted mean silhouette = 0.832.*

| **Cluster ID** | **Label** | **Silhouette** | **Size** | **Mean Year** | **Peak Year** | **Active Period** |
| --- | --- | --- | --- | --- | --- | --- |
| 0 | biomarkers | 0.89 | 5 | 2022.0 | 2022 | 2022-2024 |
| 1 | gut microbiota | 0.76 | 21 | 2024.0 | 2024 | 2019-2025 |
| 2 | immunotherapy | 0.85 | 18 | 2023.0 | 2023 | 2018-2025 |
| 3 | colorectal cancer | 0.82 | 15 | 2023.0 | 2023 | 2019-2025 |
| 4 | prediction model | 0.91 | 12 | 2024.0 | 2024 | 2022-2025 |
| 5 | fecal microbiota | 0.78 | 14 | 2022.0 | 2022 | 2018-2024 |
| 6 | metabolites | 0.84 | 11 | 2023.0 | 2023 | 2020-2025 |
| 7 | clinical trial | 0.92 | 10 | 2024.0 | 2024 | 2023-2025 |
| 8 | probiotics | 0.79 | 9 | 2022.0 | 2022 | 2019-2024 |
| 9 | immune cells | 0.81 | 8 | 2023.0 | 2023 | 2020-2025 |
| 10 | antibiotics | 0.77 | 7 | 2021.0 | 2021 | 2018-2023 |
| 11 | mouse model | 0.86 | 6 | 2020.0 | 2020 | 2017-2022 |
| 12 | machine learning | 0.94 | 5 | 2024.0 | 2024 | 2023-2025 |
| 13 | T cells | 0.80 | 7 | 2021.0 | 2021 | 2018-2023 |
| 14 | short-chain fatty acids | 0.83 | 6 | 2022.0 | 2022 | 2019-2024 |
| 15 | bioinformatics | 0.88 | 4 | 2023.0 | 2023 | 2022-2025 |

**Table S4. Keyword Time Zone Evolution Statistics (Supporting Figure 11)**

| **Year** | **New Keywords (n)** | **Representative New Keywords** |
| --- | --- | --- |
| 2010 | 5 | immunotherapy, PD-1, CTLA-4, dendritic cells, tumor microenvironment |
| 2011 | 3 | T cells, melanoma, B cells |
| 2012 | 4 | gut microbiota, microbiome, ipilimumab, colorectal cancer |
| 2013 | 3 | anti-PD-1, nivolumab, regulatory T cells |
| 2014 | 2 | fecal microbiota transplantation, Bacteroides |
| 2015 | 5 | pembrolizumab, immune checkpoint inhibitors, dysbiosis, antibiotics, Bifidobacterium |
| 2016 | 4 | Akkermansia muciniphila, probiotics, metagenomics, NLRP3 |
| 2017 | 3 | short-chain fatty acids, butyrate, Th17 |
| 2018 | 4 | cancer immunotherapy, tumor mutational burden, NSCLC, hepatocellular carcinoma |
| 2019 | 3 | CAR-T, neoadjuvant, TNBC |
| 2020 | 3 | COVID-19, cytokine storm, lung cancer |
| 2021 | 4 | ferroptosis, cuproptosis, spatial transcriptomics, metabolomics |
| 2022 | 3 | exosomes, tertiary lymphoid structures, epigenetic regulation |
| 2023 | 3 | machine learning, scRNA-seq, pyroptosis |
| 2024 | 3 | large language model, GPT, artificial intelligence |
| 2025 | 2 | digital twins, organoid co-culture |

**Table S5. Keyword Cluster Peak Landscape Metrics (Supporting Figure 12)**

| **Cluster ID** | **Label** | **Peak Year** | **Peak Citations** | **Active Period** |
| --- | --- | --- | --- | --- |
| 0 | biomarkers | 2022 | 78 | 2020-2024 |
| 1 | gut microbiota | 2024 | 312 | 2016-2025 |
| 2 | immunotherapy | 2023 | 245 | 2018-2025 |
| 3 | colorectal cancer | 2023 | 189 | 2019-2025 |
| 4 | prediction model | 2024 | 156 | 2021-2025 |
| 5 | fecal microbiota | 2022 | 134 | 2018-2024 |
| 6 | metabolites | 2023 | 98 | 2020-2025 |
| 7 | clinical trial | 2024 | 142 | 2022-2025 |
| 8 | probiotics | 2022 | 87 | 2019-2024 |
| 9 | immune cells | 2023 | 76 | 2020-2025 |
| 10 | antibiotics | 2021 | 65 | 2018-2023 |
| 11 | mouse model | 2020 | 54 | 2017-2022 |
| 12 | machine learning | 2024 | 89 | 2022-2025 |
| 13 | T cells | 2021 | 72 | 2018-2023 |
| 14 | short-chain fatty acids | 2022 | 58 | 2019-2024 |
| 15 | bioinformatics | 2023 | 45 | 2021-2025 |
